# Supplementary material for: A Novel Synthesized 1D Nanobelt-like Cobalt Phosphate Electrode Material for Excellent Supercapacitor Applications
Source: Materials (Basel). 2022 Nov 19;15(22):8235. doi: 10.3390/ma15228235 (PMC9698180; doi:10.3390/ma15228235)
Supplement: Supplementary file 1 [file materials-15-08235-s001.zip › materials-1958879-supplementary.pdf]

A Novel Synthesized 1D Nanobelt-like Cobalt Phosphate Electrode Material for Excellent Supercapacitor Applications

S. K. Shinde [1,*], Monali B. Jalak [2], Swapnil S. Karade [3], Sutripto Majumder [4], Mohaseen S. Tamboli [5], Nguyen Tam Nguyen Truong [6,*], Nagesh C. Maile [7], Dae-Young Kim [1], Ajay D. Jagadale [8] and H. M. Yadav [9]

1 Department of Biological and Environmental Science, College of Life Science and Biotechnology, Dongguk University, Biomedical Campus, 32 Dongguk-ro, Ilsandong-gu, Siksa-dong, Goyang-si 10326, Republic of Korea
2 Department of Physics, Shivaji University, Kolhapur 416004, India
3 Department of Green Technology, University of Southern Denma.+8/rk, Campusvej 55, DK-5230 Odense, Denmark
4 Department of Physics, Yeungnam University, Gyeongsan 38541, Republic of Korea
5 Korea Institute of Energy Technology (KENTECH), 200 Hyeokshin-ro, Naju 58330, Republic of Korea
6 School of Chemical Engineering, Yeungnam University, 280 Daehak-Ro, Gyeongsan 38541, Republic of Korea
7 Department of Environmental Engineering, Kyungpook National University, 80 Daehak-ro, Buk-gu, Daegu 41566, Republic of Korea
8 Center for Energy Storage and Conversion, School of Electrical & Electronics Engineering, SASTRA Deemed University, Thanjavur 613401, India
9 School of Nanoscience and Biotechnology, Shivaji University, Kolhapur 416004, India
* Correspondence: surendraphy09@gmail.com (S.K.S.); tamnguyentn@ynu.ac.kr (N.T.N.T.)

Supportting information

Figure S1 (a-d) TEM images of the $Co_2P_2O_7$ materials synthesized at deposition temperatures of 120 ^{0}C, respectively, (e) SAED pattern, (f-i) elemental mapping of the optimized $Co_2P_2O_7$ materials, respectively.

Figure S2 (a, b) TEM images of the $Co_2P_2O_7$ materials synthesized at deposition temperatures of 180 ^{0}C, respectively, (c) SAED pattern, (d-g) elemental mapping of the optimized $Co_2P_2O_7$ materials, respectively.

Figure S3 Retention capacity of the CoP-120, CoP-150 and CoP-180 electrodes upto 4000 cycles.

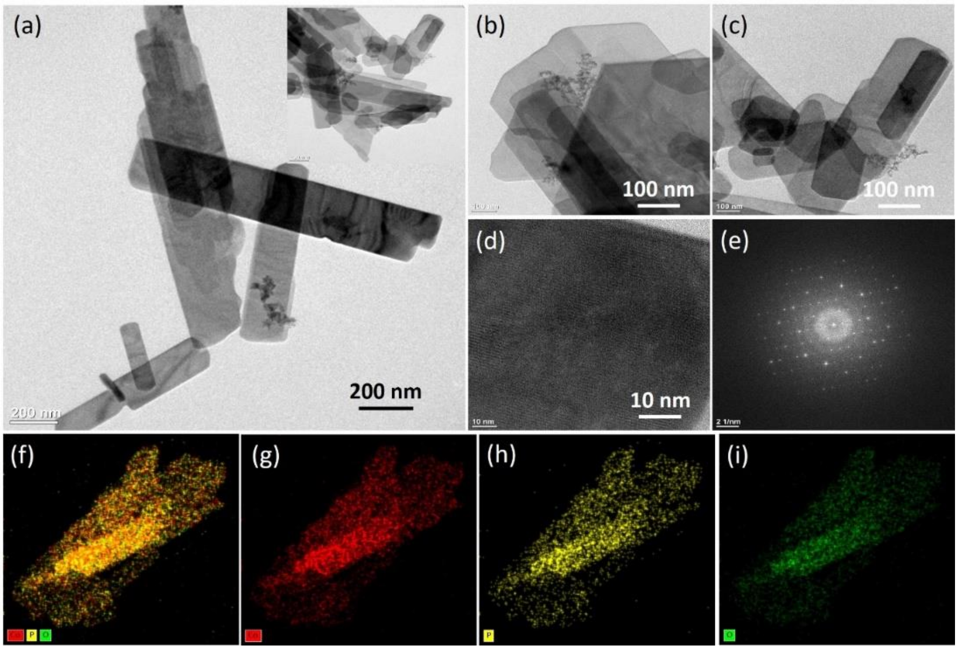

Figure S1

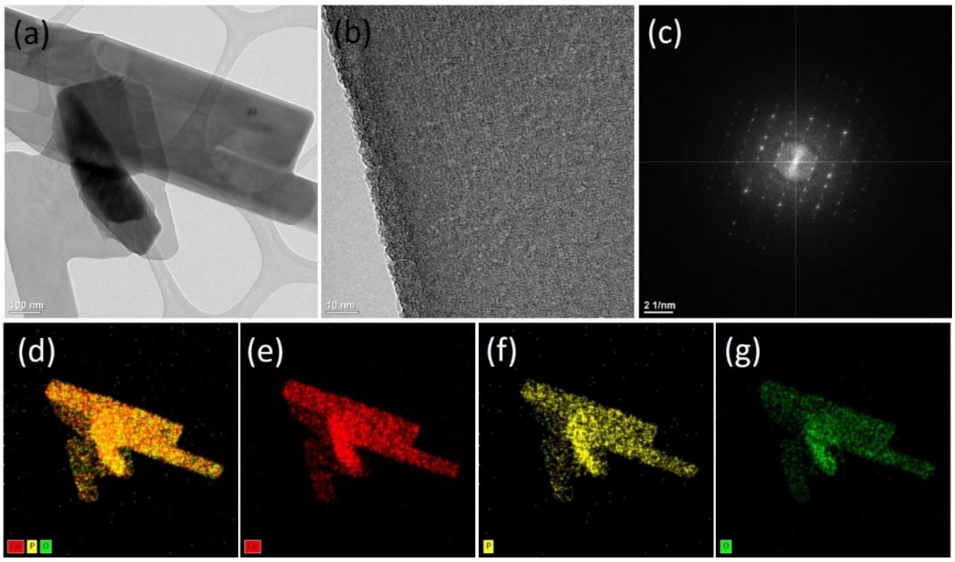

Figure S2

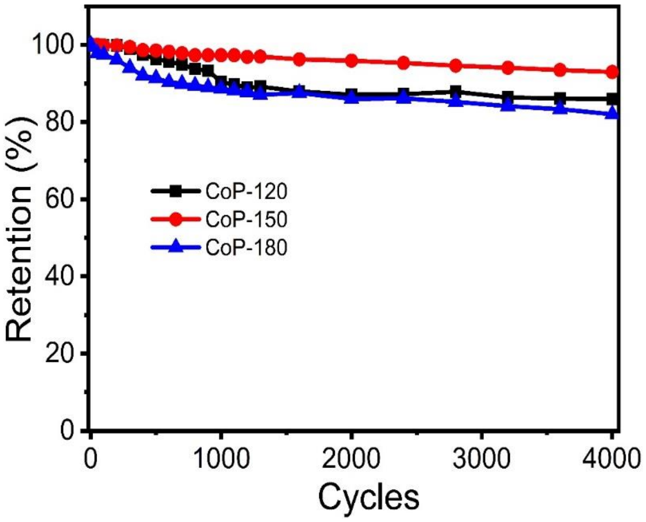

Figure S3
